# Seroepidemiology of Selected Arboviruses in Febrile Patients Visiting Selected Health Facilities in the Lake/River Basin Areas of Lake Baringo, Lake Naivasha, and Tana River, Kenya

**DOI:** 10.1089/vbz.2014.1686

**Published:** 2015-02-01

**Authors:** Caroline Tigoi, Olivia Lwande, Benedict Orindi, Zephania Irura, Juliette Ongus, Rosemary Sang

**Affiliations:** ^1^ International Centre of Insect Physiology and Ecology, Nairobi, Kenya.; ^2^ College of Health Sciences, Jomo Kenyatta University of Agriculture and Technology, Nairobi, Kenya.; ^3^ Ministry of Public Health and Sanitation, Kenyatta National Hospital grounds, Nairobi, Kenya.; ^4^ School of Public Health, Katholieke Universiteit Leuven, Leuven, Belgium.; ^5^ Centre for Virus Research, Kenya Medical Research Institute, Nairobi, Kenya.; ^6^ Department of Clinical Microbiology, Umeå University, Sweden.

**Keywords:** Arboviruses, Crimean–Congo hemorrhagic fever virus, Rift Valley fever virus, West Nile virus, Chikungunya virus, Febrile patients, Lake/River Basin areas, Kenya

## Abstract

***Introduction:*** Arboviruses cause emerging and re-emerging infections affecting humans and animals. They are spread primarily by blood-sucking insects such as mosquitoes, ticks, midges, and sandflies. Changes in climate, ecology, demographic, land-use patterns, and increasing global travel have been linked to an upsurge in arboviral disease. Outbreaks occur periodically followed by persistent low-level circulation.

***Aim:*** This study was undertaken to determine the seroepidemiology of selected arboviruses among febrile patients in selected lake/river basins of Kenya.

***Methods:*** Using a hospital-based cross-sectional descriptive survey, febrile patients were recruited and their serum samples tested for exposure to immunoglobulin M (IgM) and IgG antibodies against Crimean–Congo hemorrhagic fever virus (CCHFV), Rift Valley fever virus (RVFV), West Nile virus (WNV), and chikungunya virus (CHIKV). Samples positive for CHIKV and WNV were further confirmed by the plaque reduction neutralization test (PRNT).

***Results:*** Of the 379 samples examined, 176 were IgG positive for at least one of these arboviruses (46.4%, 95% confidence interval [CI] 41.4–51.5%). Virus-specific prevalence for CCHF, RVF, WN, and CHIK was 25.6%, 19.5%, 12.4%, and 2.6%, respectively. These prevalences varied significantly with geographical site (*p*<0.001), with Tana recording the highest overall arboviral seropositivity. PRNT results for Alphaviruses confirmed that the actual viruses circulating in Baringo were Semliki Forest virus (SFV) and CHIKV, o'nyong nyong virus (ONNV) in Naivasha, and SFV and Sindbis virus (SINDV) in Tana delta. Among the flaviviruses tested*,* WNV was circulating in all the three sites.

***Conclusion:*** There is a high burden of febrile illness in humans due to CCHFV, RVFV, WNV, and CHIKV infection in the river/lake basin regions of Kenya.

## Introduction

Arboviruses are among the most important emerging infectious diseases facing the world today; they are spread primarily by blood-sucking insects (Gubler 2002). Changes in climate, ecology, demographic, land-use patterns, and increasing global travel have been linked to an upsurge in arboviral disease (Gould and Higgs 2009, Weaver and Reisen 2010). Environmentally driven outbreaks like Rift Valley fever (RVF) in East Africa was closely associated with the heavy rainfall that occurred during the El Niño Southern Oscillation (ENSO) phenomenon in 1997/98 and 2006/07 and also by human activity, e.g., irrigation in West Africa (Woods et al. 2002, Nguku et al. 2010). Viruses such as chikungunya virus (CHIKV) have undergone genetic transformation to cause more extensive outbreaks in Indian Ocean and India (Schuffenecker et al. 2006). Outbreaks of CHIKV were reported in the Democratic Republic of Congo (1999–2000), Gabon (2007), Indian Ocean (2005–2006), India (2006–2007), Italy (2007), Thailand (2009), Sierra Leone (2012), Cambodia (2012), and the Caribbean (2013–2014) (World Health Organization Report 2014).

Outbreaks occur periodically across Kenya, but limited surveillance takes place during interepidemic periods. Studies have shown evidence of interepidemic and postepidemic transmission of RVFV among humans ranging from 1% to 33% in different settings in Kenya (LaBeaud et al. 2007, 2008). Other arboviral infections have been documented from past serosurveys, including West Nile virus (WNV), CHKV, dengue virus (DENV), o'nyong nyong virus (ONNV), and yellow fever virus (YFV) (Sutherland et al. 2011). The low-level circulation that persists during these periods has never been fully quantified due to limited surveillance and lack of proper diagnostic tools that can accurately detect these viruses (LaBeaud et al. 2011a). The public health importance of these diseases is mainly appreciated during an outbreak by governments due to the severity of symptoms caused. Most recently, Kenya has experienced multiple dengue fever outbreaks since February, 2013, to date in Mombasa and Mandera (MOH Quarterly Bulletin, June 2013; Kenya Medical Research Institute [KEMRI]; unpublished lab reports 2014). The absence of effective vaccines and therapeutic treatment for most arboviral infections underscores the need for active surveillance to monitor circulation and to inform public health decisions for early warning and response.

A number of important arboviral infections such as WNV and RVF have been isolated from mosquito vectors in Kenya (LaBeaud et al. 2011b, Sang et al. 2011, Ochieng et al. 2013). However, the true prevalence of arbovirus circulation among human populations in Kenya remains unknown, and determination of etiologies of febrile illnesses continues to present a challenge. Some of the undiagnosed febrile illnesses in health facilities could be attributed to arboviral activity. The present study aimed at determining the seroepidemiology and associated risk factors of selected arboviruses among febrile patients in the lake/river basin areas of Baringo, Naivasha, and Tana, in Kenya.

## Materials and Methods

### Study design and sites

**FIG. 1.**
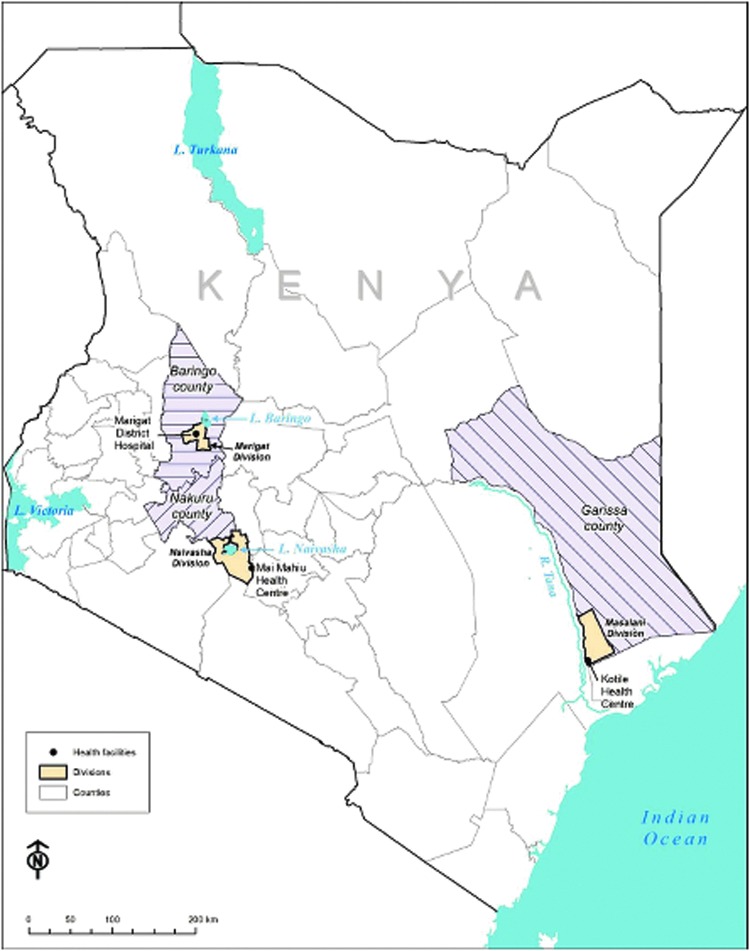
Map of Kenya showing location of the study sites in Baringo, Naivasha, and the Tana.

This was a hospital-based, cross-sectional, descriptive survey conducted between September, 2009, and December, 2012. The study was conducted at three selected health facilities: Marigat District Hospital in Baringo, Maai Mahiu Health Centre in Naivasha, and Kotile Health Centre in Tana River districts of Kenya (Fig. 1). There are similarities in environmental variables in these sites that are likely to promote active transmission of arboviral infections, including low rainfall, flood-prone basins, increased temperatures, proximity to rivers or lakes, forested areas, and presence of wildlife and several bird species. Movement by the nomadic pastoralist communities in search of pasture and water in addition to availability of competent vectors (e.g. *Hyalomma* spp. of ticks, *Aedes, Anopheles,* Mansonia, and *Culex,* species of mosquitoes) may facilitate transmission and exposure to arboviral infection in these ecosystems.

Baringo district (latitude 0.28°N and longitude 35.58°E) and Naivasha (latitude 0.72°S and longitude 36.43°E) in Rift Valley province have populations of 173,815 and 225,547, respectively. Tana basin (latitude 2°32′S and longitude 40°31′E) borders Ijara district in North Eastern Province and has a population of 164,115 (Kenya National Bureau of Statistics 2009).

### Study population

The study population comprised persons (male and female) aged ≥5 years attending the three health facilities (one in each area) selected on the basis of their proximity to the lake/river basin areas as well as the number of reported unexplained febrile patients being seen at the facility on a daily basis. The patients presenting with a clinical case definition of acute febrile illness characterized by fever of temperature ≥38°C and with at least one of the following clinical manifestations: Cough, joint pains, headache, chills, general body malaise, and any signs of bleeding were recruited into the study. A patient was excluded from the study if no assent or consent was provided to participate in the study.

### Sample collection

A total volume of 5 mL of blood was collected from the study participants using Vacutainer tubes with a clot activator. Serum was processed by centrifugation at 1500 rpm for 10 min. The serum was aliquoted into 1-mL volumes, placed in sterile bar-coded cryovials, and stored in liquid nitrogen at the health facilities until collection and transportation in dry ice to the KEMRI laboratories, where they were stored at −70°C, processed, and tested.

### Laboratory testing procedures

#### Immunoglobulin M capture and indirect IgG capture enzyme-linked immunosorbent assay

All of the serum samples brought to the laboratory were heat inactivated at 56°C for 30 min and screened for exposure to Crimean–Congo hemorrhagic fever virus (CCHFV), CHIKV, WNV, and RVFV using immunoglobulin M (IgM) and IgG capture enzyme-linked immunosorbent assay (ELISA) (for CCHFV and CHIKV) and IgM and IgG indirect ELISA (for WNV and RVFV). The commercial kits used were manufactured by Vector Best for CCHFV (www.vector-best.ru), BDSL, United Kingdom, for RVFV, Novatec Immunodiagnostica GMBH for CHIKV, and Panbio, Germany, for WNV. These tests were conducted strictly based on manufacturer's instructions.

### Plaque reduction neutralization test

Samples positive for CHIKV and WNV antibodies by IgG ELISA were further tested using a plaque reduction neutralization text (PRNT) to rule out cross-reactivity with other alphaviruses and flaviviruses known to be endemic in the area, specifically Semliki Forest virus (SFV), Sindbis virus (SINDV), ONNV, DENV, Uganda S virus (UGSV), and YFV. Each virus isolate was diluted to a standard concentration that produced approximately 20–50 plaques. Serum samples were heat inactivated at 56°C for 30 min. Each sample was serially diluted in a sterile 96-well plate to determine the end point titer or highest dilution that neutralizes at least 99% of the virus at 1:20 to 1:640 concentration in maintenance medium (minimum essential media [Sigma] with Earle's salts, 2% fetal bovine serum, 2% glutamine, 100 U/mL penicillin, 100 μg/mL streptomycin, and 1 μL/mL amphotericin B). A constant amount of diluted virus was added into each well of the 96-well plate containing serially diluted serum samples and incubated for 1 h at 37°C. The virus–antibody mixture was then transferred to a 24-well plate with a confluent Vero cell monolayer and incubated in CO_2_ for 1 h for virus adsorption, after which an overlay of 2.5% methylcellulose was added and incubated for 5–10 days. The plates were retrieved from the incubator and stained with 0.25% Crystal Violet in absolute ethanol. A PRNT_99_ antibody titer for each virus was required to be four-fold greater than the other viruses in the family tested to make an etiological diagnosis.

### Data analysis

Field data captured in the study questionnaires were stored in a password-protected database linked to the laboratory results, and the data were analyzed using Stata v. 10.1 (StataCorp, College Station, TX). The main outcomes of interest were IgG antibodies against CCHFV, CHIKV, WNV, and RVFV. The IgG status was binarized as either positive or negative based on the optical density (OD) values. Data on prevalence were compared using chi-square or Fisher exact test. The 95% confidence intervals (CIs) for the proportions positive for a virus were estimated using the Agresti–Coull method (Brown et al. 2001). We performed logistic regression analyses to understand the factors predictive of the risk of infection by each of the four viruses among the people in the study areas. The factors considered included site, age of the subjects, gender, occupation, and contact with animals and/or birds, whether or not a subject had tick-bites, and whether the subjects made contact through farming or through slaughter. All tests were performed at 5% significance level.

### Ethical considerations

The study was approved by Kenya Medical Research Institute's Scientific Steering Committee and Ethical Review Committee (SSC no. 2350). Informed consent was obtained from all enrolled patients.

## Results

### Demographic and clinical characteristics of the study participants

In total, 379 human serum samples were collected from patients presenting with febrile illness, including fevers of unknown origin at health facilities in Naivasha (*n*=152), Baringo (*n*=117), and Tana (*n*=110). The mean/median age for the participants was 24.4 years/22 years and the age range was 5–80 years. Ten individuals did not give their age details for unknown reasons. The ages were further classified into four age categories of size 10. Because those aged below 10 years were few (*n*=57), they were lumped into a single age category with those aged 10–19 years. The results indicated that most of the subjects (84%) were less than 40 years old. Of the total participants, 224 (59%) were females and 155 (41%) males (Table 1).

**Table 1.** Sociodemographic Characteristics of Study Participants by Site

	*Site*			
*Characteristic*	*Naivasha* n*=152*	*Baringo* n*=117*	*Tana* n*=110*	*Total* n*=379*
Sex				
Female	88	71	65	224
Male	64	46	45	155
Age				
5–19	33	83	32	148
20–29	66	18	25	109
30–39	28	5	27	60
40+	20	6	26	52
Missing	5	5	0	10
Tick bite				
No	139	52	110	301
Yes	13	65	0	78
Contact with donkey				
No	68	111	93	272
Yes	84	6	17	107
Contact with goats				
No	55	9	65	129
Yes	97	108	45	250
Contact with cows				
No	55	41	79	175
Yes	97	76	31	204
Contact with ducks				
No	132	117	109	358
Yes	20	0	1	21
Made contact through farming				
No	70	48	108	226
Yes	82	69	2	153
Made contact through slaughter				
No	138	117	110	365
Yes	14	0	0	14
Occupation				
Teaching/schooling	35	87	26	148
Business	40	3	5	48
Casual worker	17	7	9	33
Farmer	29	7	18	54
Herdsman	9	4	8	21
House Wife	22	9	44	75

Seventy-six percent of the patients reported to have had contact with animals and 66% with birds; 21% reported to have suffered from tick bites. They presented with the following symptoms: Fever (100%), headache (91.03%), joint aches (75.99%), muscle aches (69.93%), chills (64.64%), abdominal pain (57.52%), cough (45.91%), sore throat (36.15%), and vomiting (35.09%).

### IgM and IgG seroprevalence results for CCHFV, CHIKV, WNV, and RVFV

**FIG. 2.**
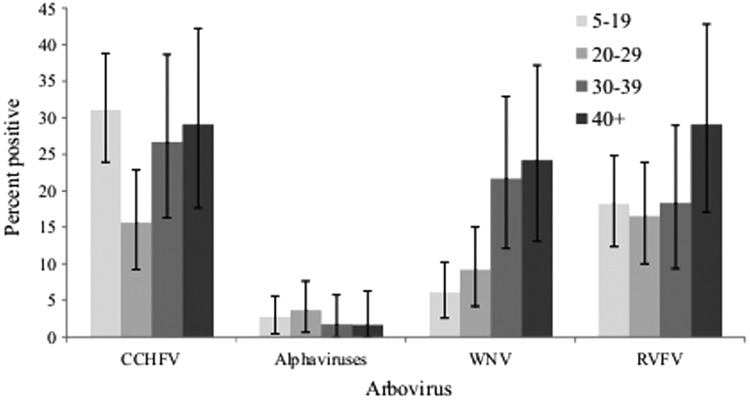
Immunoglobulin G (IgG) prevalence of Crimean–Congo hemorrhagic fever virus (CCHFV), *Alphavirus*, West Nile virus (WNV), and Rift Valley fever virus (RVFV) by age group. The error bars indicate Agresti–Coull 95% confidence intervals.

None of the subjects was positive for anti-CCHFV, anti-WNV, and anti-RVF IgM. An acute case of anti-CHIKV IgM was detected in Tana (1/379). A total of 176 (46.44%, 95% CI 41.39–51.48%) patients had IgG antibodies against at least one of the four arboviruses. The prevalences of each virus calculated separately and for all four viruses combined by site are presented in Figure 2 and Table 2.

**Table 2.** ELISA Anti-Arboviral IgG Antibodies Prevalence (Number Positive) Recorded Naivasha, Baringo, and Tana for Each Virus Separately and Combined

*Variable*	*CCHFV*	*Alphavirus*	*WNV*	*RVFV*	*All arbovirus combined*
All sites	25.59 (97)	2.64 (10)	12.40 (47)	19.53 (74)	46.44 (176)
Site					
Naivasha	17.76 (27)	1.97 (3)	2.63 (4)	13.82 (21)	32.24 (49)
Baringo	35.04 (41)	2.56 (3)	5.13 (6)	21.37 (25)	52.14 (61)
Tana	26.36 (29)	3.64 (4)	33.64 (37)	25.45 (28)	60.00 (66)
*p* value	0.005	0.667	<0.001	0.053	<0.001

ELISA, enzyme-linked immunosorbent assay; IgG, immunoglobulin G; CCHFV, Crimean–Congo hemorrhagic fever virus; WNV, West Nile virus; RVFV, Rift Valley fever virus.

The anti-CCHFV IgG prevalence was 25.6% (95% CI 21.2–30.0). The prevalence of CCHFV varied by site (*p*<0.001), and it was the most prevalent virus in all the three sites. The prevalence was highest among those aged 5–19 and 40+ years old at 31.08% and 28.85%, respectively. The prevalence among those who had contact with animals (did not have contact with animals) was 28.47% (16.48%).

The overall IgG prevalence for alphaviruses was 2.6% (95% CI 1.0–4.3%). There was no statistically significant difference in alphavirus prevalence among the sites (*p*=0.667). Of the 10 samples positive for alphaviruses by ELISA, one (10%) neutralized with CHIKV, five (50%) with SFV, three (30%) with ONNV, and one (10%) with SINDV. The overall prevalences of CHIKV and SINDV confirmed by PRNT were both 0.26%, whereas the prevalence of SFV was 1.3% and ONNV was 0.8% (Table 3).

**Table 3.** PRNT Results for *Alphavirus* and *Flavivirus* Genera

	*Alphavirus family*			
*Site*	*SFV*	*CHIKV*	*SINV*	*ONV*
Naivasha	0 (0.0)	1 (0.7)	0 (0.0)	0 (0.0)
Baringo	2 (1.7)	0 (0.0)	0 (0.0)	3 (2.6)
Tana	3 (2.7)	0 (0.0)	1 (0.9)	0 (0.0)

	*Flavivirus genus*			
*Site*	*UGSV*	*DENV*	*WNV*	*YFV*
Naivasha	0 (0.0)	0 (0.0)	4 (2.6)	0 (0.0)
Baringo	0 (0.0)	0 (0.0)	6 (5.1)	0 (0.0)
Tana	0 (0.0)	0 (0.0)	37 (33.6)	0 (0.0)

PRNT, plaque reduction neutralization test; SFV, Semliki Forest virus; CHIKV, chikungunya virus; SINV, Sindbis virus; ONNV, O'nyong nyong virus; UGSV, Uganda S virus; DENV, Dengue virus; WNV, West Nile virus; YFV, yellow fever virus.

The IgG prevalence for WNV was 12.4% (95% CI 9.10–15.70%). The prevalence of WNV varied by site (*p*<0.001) and increased with age. Those aged 40 years and above were more likely to be exposed than the younger populations (Fig. 2 and Table 4). All of the samples positive for WNV by ELISA were confirmed to be WNV by PRNT, recording an overall prevalence of 12.4% (Table 3).

**Table 4.** Risk Factors for Crimean–Congo Hemorrhagic Fever Virus, Alphaviruses, West Nile Virus, and Rift Valley Fever Valley: Multiple Logistic Regression Model Results

	*OR (95% CI)*			
*Variable*	*CCHFV*	*Alphaviruses*	*WNV*	*RVF*
Site				
Naivasha	1.00	1.00	1.00	1.00
Baringo	1.57 (0.76–3.23)	1.46 (0.25–8.57)	2.83 (0.68–11.81)	**2.11 (1.00–4.45)**
Tana	1.84 (0.96–3.51)	2.27 (0.47–10.96)	**18.26 (5.94–56.12)**	**2.47 (1.11–5.52)**
Gender: Male	0.97 (0.59–1.59)	2.31 (0.63–8.43)	1.66 (0.81–3.42)	0.72 (0.42–1.25)
Age-group (years)				
5–19	1.00	1.00	1.00	1.00
20–29	**0.48 (0.24–0.96)**	1.66 (0.36–7.64)	2.11 (0.75–5.97)	1.17 (0.57–2.42)
30–39	1.07 (0.50–2.26)	0.62 (0.06–6.29)	**3.55 (1.26–10.00)**	1.21 (0.51–2.84)
40+	1.14 (0.53–2.46)	0.61 (0.06–6.11)	**4.12 (1.47–11.51)**	**2.63 (1.18–5.85)**
Tick bite	1.00 (0.47–1.89)			
Contact with goat	3.38 (1.68–6.80)			
Contact with ducks			2.10 (0.16–27.87)	
Type of contact made				
Farming				1.44 (0.73–2.84)
Slaughter				1.05 (0.21–5.33)

OR, odds ratio; CI, confidence interval; CCHFV, of Crimean–Congo hemorrhagic fever virus; WNV, West Nile virus; RVF, Rift Valley virus.

The overall IgG prevalence for RVFV was 19.5% (95% CI 15.5–23.5%). There was a borderline significant association between RVFV prevalence and site (*p*=0.053). Its prevalence increased with age (Table 4). Of the 176 subjects who were positive for at least one arbovirus, 133 had a single infection, 34 double infections, and nine had triple infections. One sample was positive for both SINDV and WNV by PRNT.

### Risk factors associated with CCHFV, CHIKV, WNV, and RVFV infection

The logistic regression analysis results for each virus are summarized in Table 4. Compared to Naivasha and after controlling for the other factors, the results showed that the odds of CCHFV infection were similar in the three sites. The odds of infection were, however, higher in females than in males but not statistically significant. The results further indicated that those who had contact with goats were three times more likely to be infected with CCHFV than those who did not (odds ratio [OR]=3.38, 95% CI 1.68–6.80). Occupation was not significant for CCHFV as well as the other three viruses.

There was no difference in odds of being infected with an alphavirus by site. Infection was seen to be higher among males. Compared to the first age group, the odds of being infected were higher among those aged between 20–29 years, but not statistically significant.

Compared to Naivasha, the odds of infection with WNV were higher in Tana (OR=18.26, 95% CI 5.94–56.12). The results also showed that the risk of infection by WNV increased significantly with age and was higher in males compared to females. Relative to 5–19 years and after adjusting for other factors, those in the age groups 20–29 years were almost twice (OR=2.11, 95% CI 0.75–5.97), 30–39 years were three times (OR=3.55, 95% CI 1.26–10.00), and 40+ years were four times (OR=4.12, 95% CI 1.47–11.51) more likely to be infected by WNV.

The odds of infection with RVFV were higher in Baringo (OR=2.11, 95% CI 1.00–4.45) and Tana (OR=2.47, 95% CI 1.11–5.52) as compared to Naivasha. These odds were also higher in females than males, but the difference was not statistically significant. Those aged 40+ years were more likely to be infected by RVFV (OR=2.63, 95% CI 1.18–5.85) than those aged 5–19 years. Those who made contact with animals and/or birds through farming and slaughtering had 44% (OR=1.44, 95% CI 0.73–2.84) and 5% (OR=1.05, 95% CI 0.21–5.33), respectively, higher (but not statistically significant) odds of being infected by RVFV than those who did not make any contact with animals and/or birds through farming and slaughtering (Table 4).

## Discussion

This study focused on habitats with similar ecological, environmental, and biological factors to quantify the burden of disease among the human population whose main source of livelihood is pastoralism. The overall prevalence of arboviral infection varied across the sites, with Tana recording the highest prevalence. The high risk in Tana can be attributed to environmental modification by the river with flood plains formed during rains and increased temperatures that favor breeding of mosquitoes that transmit arboviruses. The convergence of wildlife, migratory birds, and livestock for water and pasture, especially during the dry seasons, leads to exposure to the vectors. Tana is a home for many bird species (*e.g*., Passeriformes) that have been implicated as a preferred avian host for SINV and WNV (Buckley et al. 2003, Komar et al. 2003). This finding has public health implications because prevention programs should be site specific and not country wide.

The study showed that CCHFV was the most prevalent virus in all the sites with an overall prevalence of 25.6%. This is close to 19% prevalence obtained in a study by Lwande et al. (2012) in Ijara District, Kenya. Circulation of the virus in humans is expected given that this virus has been isolated previously from *Hyalomma* spp. of ticks in Kenya (Sang et al. 2011). Being a female and being in contact with goats increased the risk of exposure to CCHFV. A higher risk of CCHFV among those who had contact with goats can be attributed to the fact that goats are heavily infested by ticks that can bite humans when they handle the goats. This finding agrees with that of Sargianou et al. (2013). Prevalence was highest among those aged 5–19 and 40+ years old that partly compares well with the findings by Lwande et al. (2012), where CCHFV was most prevalent among those aged between 40 and 49 years. This could be attributed to the age group being in close and frequent contact with animals during herding, high-risk occupations like butchers, physicians, and veterinarians or during trade exposing them to infection. The age group 5–19 years and females could be exposed during lambing or milking within the homestead where they spend most of their time. The middle age group 20–29 years was at a significantly lower risk of infection compared to the younger age group (*i.e*., 5–19). This is an interesting finding because other studies have shown an increase in infection with age (Fisher-Hoch et al. 1992, Lwande et al. 2012). This finding needs further investigation of the risk factors associated with infection among the younger age group not investigated in this study. This observation can, however, be attributed to the middle-aged persons being most active in society at school and colleges or engaged in formal employment away from home and hence less exposure to tick bites and infected livestock.

The odds of infection with CCHFV in Baringo were higher compared to Naivasha, probably because of geographic, environmental, and cultural differences in the two sites. Baringo is more arid compared to Naivasha, with most of its population practicing nomadic pastoralism. It experiences warmer temperatures and is close to two big lakes, namely Lake Baringo and Lake Bogoria, providing humidity and arid conditions suitable for the *Hyalomma* ticks, the primary vector for CCHFV. There are more livestock (*e.g*., goats) in the region compared to Naivasha and hence high risk of exposure to ticks and viremic animals by the population through contact by herding, slaughtering, and tick bites. There is a big livestock market in Baringo as well as an active slaughterhouse where exposure may occur.

Alphavirus infections circulate in low levels in all the three study sites. Cross-reactivity was observed in the family with 10 samples that had tested positive for anti-CHIKV IgG ELISA neutralizing with CHIKV (1/10), SFV (5/10), ONNV (1/10), and SINDV (1/10) by PRNT. This can be attributed to the antigenic similarity among alphaviruses that results in cross-reactivity, posing a challenge in diagnosis of arboviral infection (Sutherland et al. 2011). One acute febrile patient tested positive for anti-CHIKV IgM. The patient was a male farmer from Tana aged 36 years. He presented with headache, fever (38.3°C), sore throat, abdominal pains, joint aches, muscle ache, itchy body, and with a clinical diagnosis of typhoid and malaria. The outcome of the patient is not known because there was no follow-up done on patients recruited in this cross-sectional survey. This was later confirmed by PRNT to be SFV infection. The virus is widely distributed in Africa and with an outbreak of 22 cases being reported in Central Africa republic in 1987 (Mathiot et al. 1990). Besides, multiple isolations of SFV from mosquitoes and ticks have been documented in parts of Kenya, supporting the probability of transmission of the virus to the reported case in Tana area (Crabtree et al. 2009, Lwande et al. 2013, Ochieng et al. 2013).

The prevalence of WNV increased with age with those aged 40 years and above being at a higher risk of infection than the younger populations. This finding is similar to the findings by Mease et al. (2011), where seropositivity of WNV increased with age. This suggests a possibility of exposure to infection over time, producing a cumulative increase in IgG seropositivity with age as documented by Sutherland et al. (2011). Males were also at higher risk of infection than females. This can be attributed to men being outdoors most of the time, hence exposure to mosquito vectors. An interesting finding is that the odds of infection were higher in Baringo and Tana compared to Naivasha. This can be ascribed to the similarity in environmental characteristics of the first two sites being semiarid and having higher temperatures and humidity conducive for survival of competent vectors for WNV (*Culex* spp. of mosquitoes) isolated previously from these sites (LaBeaud et al. 2011b).

The prevalence of RVFV infection also varied with site and gender, with females being at a higher risk than males. This observation contradicts previous findings where the risk of infection with RVFV varied with gender, with males being at a higher risk than females due to their direct contact with animals during herding (LaBeaud et al. 2008, 2011a). A possible explanation for these results is that women in these areas do participate in high-risk activities like taking care of livestock and small stocks like sheep and goats, by milking, and by giving hand during slaughtering. This finding, however, compares with findings from a study conducted in Lokichoggio, Kenya (LaBeaud et al. 2007). Although not statistically significant, being in contact with animals and/or birds through farming and slaughtering increased the risk RVFV infection. This agrees with studies that have revealed that domestic mammals (such as camels, donkeys, horses, and cattle), wild animals and birds are potential reservoirs of these viruses (Pak et al. 1975, Komar et al. 2003).

The logistic regression model results (Table 4) showed that the risk of RVFV infection increased with age as expected and was more pronounced in the oldest age group. This qualifies the fact that the younger age group does not make direct contact with infected animals and that there is a possibility of the elderly having been exposed over time in the previous outbreaks in 1996–1997 and 2006–2007 and hence persisting antibodies were detected (LaBeaud et al. 2011a). The high prevalence of infection by RVFV in Tana and Baringo could be due to widespread flooding during the rainy season and extended standing of water, and hence hatching of transovarially infected floodwater *Aedes* mosquitoes (Woods et al. 2002). This is a common phenomenon in Baringo and Tana, which are mostly semiarid, with soil that has high water retention capacity. The high temperatures and relative humidity in these areas also provide favorable breeding sites for mosquitoes (Labeud et al. 2007).

Cross-reactivity was observed in the *Alphavirus* genus with samples previously positive for CHIKV virus by ELISA being confirmed to have antibodies for SFV, ONNV, and SINDV. This agrees with study findings in Cameroon where cross-reactivity was observed in the *Alphavirus* family, especially in CHIKV and ONNV (Kuniholm et al. 2006). One sample neutralized with both *Alphavirus* (SINDV) and *Flavivirus* (WNV) by PRNT. Cross-reactivity among alphaviruses and flaviviruses is a common phenomenon that creates challenges in arbovirus detection and confirmation in endemic places. Co-infection with more than one virus was observed in 19% of the patients by ELISA and in one sample by PRNT. This was also reported in a study conducted in Nigeria, where 92% of the patients were co-infected with more than one arbovirus, typhoid, and/or malaria (Baba et al. 2013). The occurrence of co-infections can be due to current or past exposure to vectors transmitting the virus. Lack of sensitive diagnostic methods, as observed by other studies, can lead to underestimation of the burden arboviral prevalence in the population (Sutherland et al. 2011).

The most common mosquito-borne arbovirus in the three sites was RVFV, followed by WNV; alphaviruses were least prevalent. These viruses have been shown to be circulating during the interepidemic periods in these areas (LaBeaud et al. 2008, Mease et al. 2011). There was only one acute alphavirus case detected in the study. Previous research has shown that IgM antibodies diminish within 45 days of exposure (Madani et al. 2003). The low IgM prevalence is possible due to delayed health-seeking behavior by febrile patients due to limited resources for health care and very far distances of travel to arrive at a medical facility. Future studies can explore the use of more sensitive methods like PCR not performed in this study to detect acute febrile cases.

## Conclusion

This study suggests circulation and exposure of human populations to CCHFV, RVFV, WNV, and CHIKV in the river/lake basin areas of Baringo, Naivasha, and Tana. Underreporting of arboviral infection poses a serious public health concern in the study area due to misdiagnosis as malaria/typhoid and the presence of multiple co-infections. Efforts to recognize and identify cases should be made through awareness creation among clinicians in the affected areas. Additionally, infection control measures targeting the significant risk factors should be put in place to alleviate the burden of disease in these areas. There is a need for active surveillance and use of improved diagnostic tools to monitor circulation to inform public health decisions for early warning and response.
